# Supplementary material for: Variational Autoencoders for Cancer Data Integration: Design Principles and Computational Practice
Source: Front Genet. 2019 Dec 11;10:1205. doi: 10.3389/fgene.2019.01205 (PMC6917668; doi:10.3389/fgene.2019.01205)
Supplement: Supplementary file 1 [file Presentation_1.pdf]

Supplementary Material for: Variational Autoencoders for Cancer Data Integration

Nikola Simidjievski [1,†,*], **Cristian Bodnar** [1,†], **Ifrah Tariq** [1,2,†], **Paul Scherer** [1], **Helena Andres Terre** [1], **Zohreh Shams** [1], **Mateja Jamnik** [1] **and Pietro Liò** [1]

[1] *Department of Computer Science and Technology, University of Cambridge, Cambridge, United Kingdom*
[2] *Department of Biological Engineering, Massachusetts Institute of Technology, Cambridge, MA, USA*
[†] *Contributed equally*

Correspondence*:
Nikola Simidjievski, Department of Computer Science and Technology, University of Cambridge, 15 JJ Thomson Avenue, CB3 0FD Cambridge, United Kingdom
nikola.simidjievski@cl.cam.ac.uk

We investigated 108 different configurations for each of the four integrative architectures for each integrative task. Figure 1 and Figure 2 highlight the effect the optimal architecture designs when integrating CNA and mRNA data as well as clinical and CNA data, respectively. In particular, we investigate the size of the hidden dense layers, the size of the learned representation, the most appropriate regularisation in the objective function as well as how much this regularisation should influence the overall loss. These configurations are evaluated by comparing the average train and test performance of classifying IHC sub-typed patients.

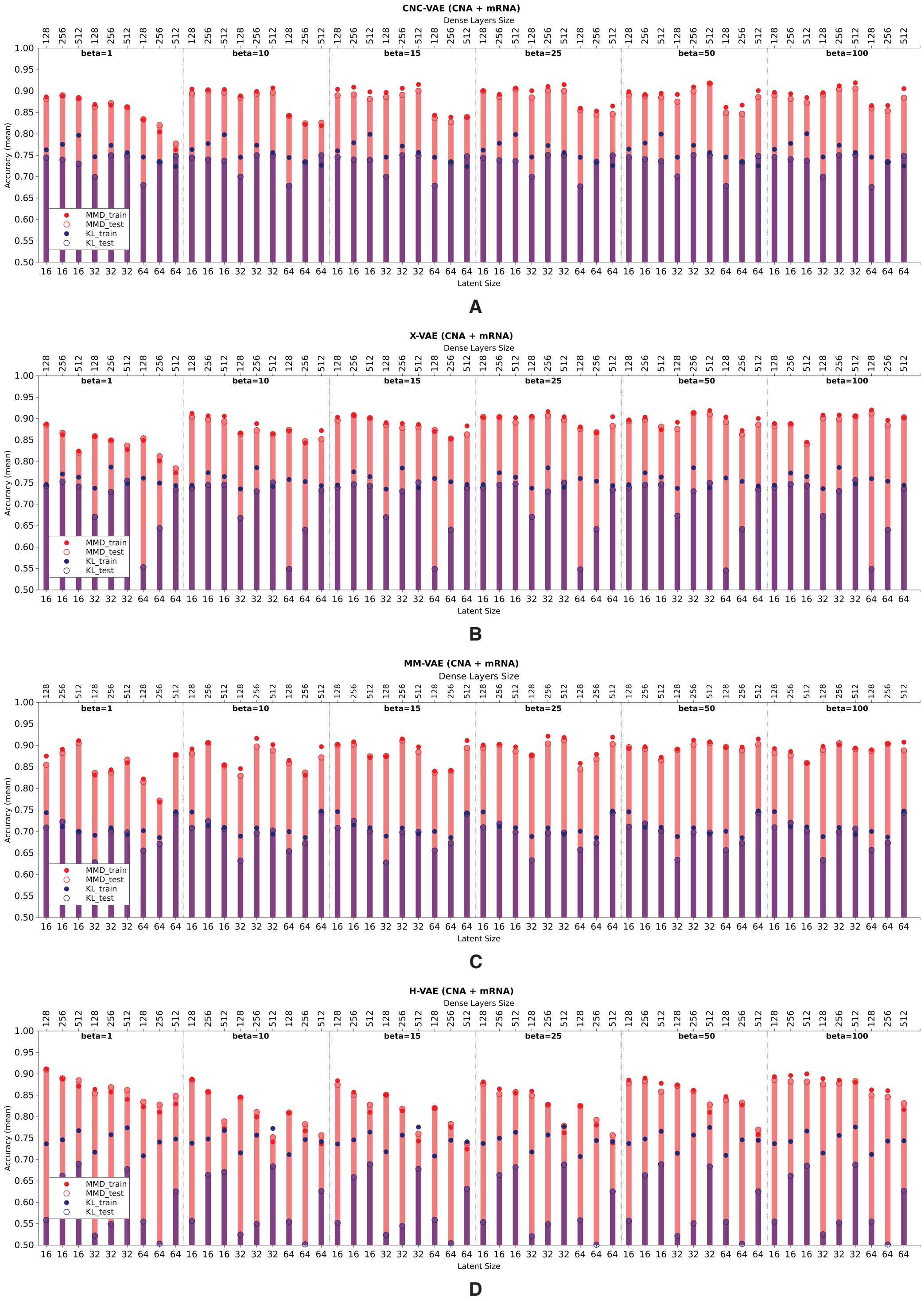

Figure 1. Comparison of the downstream performance on the IHC classification tasks of a predictive model trained on the representations produced by integrating CNA and mRNA data using (A) CNC-VAE (B) X-VAE (C) MM-VAE and (D) H-VAE. Full circles denote the training accuracy, while empty circles and bars denote the test accuracy averaged over 5-fold cross-validation. Red and blue colours denote the configurations when MMD and KL are employed, respectively. Bottom x-axis depicts the size of the latent dimension, while the top x-axis the size of the dense layers of each configuration.

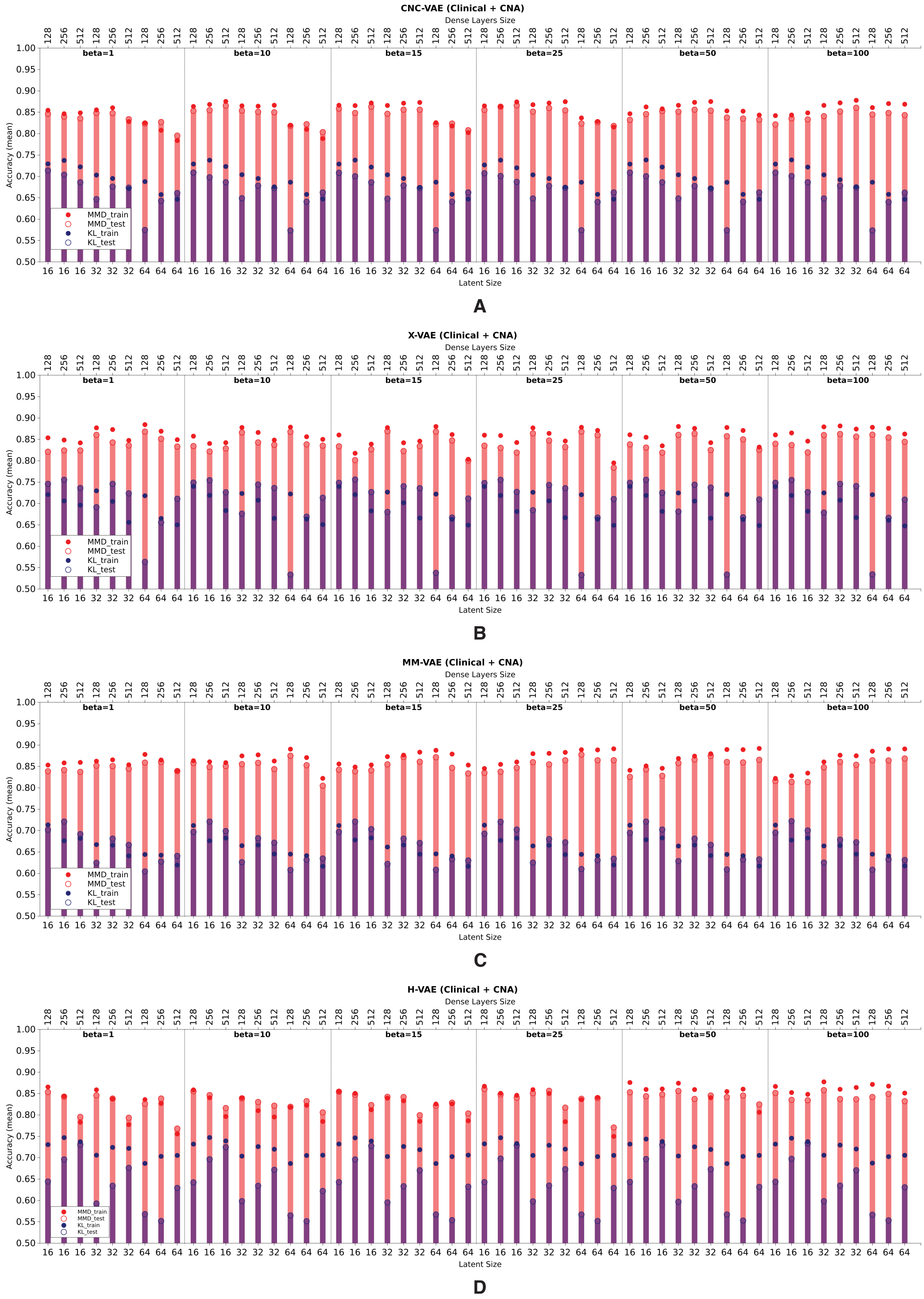

Figure 2. Comparison of the downstream performance on the IHC classification tasks of a predictive model trained on the representations produced by integrating clinical and CNA data using (A) CNC-VAE (B) X-VAE (C) MM-VAE and (D) H-VAE. Full circles denote the training accuracy, while empty circles and bars denote the test accuracy averaged over 5-fold cross-validation. Red and blue colours denote the configurations when MMD and KL are employed, respectively. Bottom x-axis depicts the size of the latent dimension, while the top x-axis the size of the dense layers of each configuration.
